# Protection-Oriented Non-Intrusive Arc Fault Detection in Photovoltaic DC Systems via Rule–AI Fusion

**DOI:** 10.3390/s26103138

**Published:** 2026-05-15

**Authors:** Lu HongMing, Ko JaeHa

**Affiliations:** Department of Electrical Engineering, Honam University, 417 Eodeung-daero, Gwangsan-gu, Gwangju 62399, Republic of Korea; luckyhm@foxmail.com

**Keywords:** DC arc fault detection, embedded systems, low-frequency sub-band selection, near-field magnetic sensing, photovoltaic (PV) systems, rule-AI fusion, shielded loop antenna

## Abstract

**Highlights:**

**What are the main findings?**

- A fully embedded, non-intrusive PV DC arc detection pipeline was implemented on a low-cost MCU using a shielded loop sensor, on-chip PGA, ADC, FFT, and a cascaded Rule–AI decision strategy.
- Across household, laboratory, and PV scenarios, the proposed method showed that broadband anomaly cues in the 12–80 kHz band can support practical arc discrimination while suppressing narrowband interference.

**What are the implications of the main findings?**

- The study supports the feasibility of low-resource, non-intrusive PV DC arc monitoring under the tested conditions.
- The proposed Z-domain normalization and Rule–AI fusion framework provides an interpretable embedded baseline for future validation under broader inverter types, environmental conditions, and installation geometries.

**Abstract:**

Series arc faults on the DC side of photovoltaic (PV) systems are a critical hazard that can trigger system fires. Conventional contact-based detection methods suffer from cumbersome installation and high retrofit cost, whereas existing non-contact approaches mostly rely on megahertz-level high-frequency sampling and therefore require expensive radio-frequency instrumentation or high-performance computing platforms. As a result, it remains difficult to simultaneously achieve strong interference immunity and real-time performance on low-cost embedded devices with limited resources. To address this engineering paradox between high-frequency sampling and constrained computational capability, this paper proposes a fully embedded, non-contact arc fault detection system based on a 12–80 kHz low-frequency sub-band selection strategy. By exploiting the physical characteristic of broadband energy elevation induced by arc faults, the proposed strategy avoids dependence on high-bandwidth hardware. Guided by this strategy, a Moebius-topology coaxial shielded loop antenna is employed as the near-field sensor, while an ultra-simplified passive analog front end is constructed directly by using the on-chip programmable gain amplifier and analog-to-digital converter of the microcontroller unit, enabling efficient signal acquisition and fast Fourier transform processing within the target sub-band. To cope with complex background noise in the low-frequency range, an environment-adaptive baseline mechanism based on exponential moving average and exponential absolute deviation is developed for dynamic decoupling. In addition, a lightweight INT8-quantized multilayer perceptron is introduced as a nonlinear auxiliary module, thereby forming a robust hybrid decision architecture with complementary rule-based and artificial intelligence components. Experimental results show that, under the tested household, laboratory, and PV-site conditions, the proposed system achieved an overall detection rate of 97%, while the remaining 3% mainly corresponded to failed ignition or non-sustained arc attempts rather than persistent false triggering during normal monitoring.

## 1. Introduction

With the evolution of photovoltaic (PV) systems toward distributed architectures and module-level power electronics (MLPE), dc series arc faults have been widely recognized as one of the major causes of fire risk [1,2,3]. Such faults are accompanied by sustained high-temperature plasma discharge and stochastic broadband electromagnetic radiation. Owing to the absence of a current zero-crossing point on the dc side, their hazard severity is considerably greater than that of ac faults. However, most existing commercial solutions still rely on intrusive current sensing. In large-scale distributed PV arrays, these approaches not only involve cumbersome installation procedures, but also suffer from high retrofit cost and the risk of single-point failure. For example, the test frameworks defined in UL 1699B and IEC 63027 generally employ thresholds such as operating time and cumulative arc energy (e.g., 2.5 s or 750 J, whichever occurs first), while also emphasizing the avoidance of false tripping under inverter noise and operating-condition variations. These requirements provide practical constraints for engineering arc-fault protection. Nevertheless, in real-world deployment, achieving an effective balance between extremely limited embedded resources in low-cost microcontroller units (MCUs) and complex electromagnetic interference (EMI) in field environments remains an open engineering challenge.

From the perspective of signal acquisition, PV dc arc-fault detection methods can generally be classified into two categories: conducted-emission-based methods and radiated-emission-based methods. Conducted-emission methods typically acquire current, voltage, or their high-frequency noise components from the cable side. Their performance is strongly affected by inverter switching noise, maximum power point tracking operating conditions, and cable parameters, causing thresholds to drift across scenarios. Radiated-emission methods, by contrast, use antennas or near-field probes to capture the electromagnetic field signals radiated by arc events. However, they are more vulnerable to background heterogeneity, sensor position, and narrowband interference sources, which makes false-alarm suppression more difficult. These two categories differ substantially in deployment mode, interference immunity, and hardware cost. In addition, auxiliary approaches based on optical, acoustic, or thermal sensing have also been explored, although their deployment in PV applications is often constrained in practice.

The conducted-measurement route is relatively mature and can be readily integrated with inverter control, but it usually requires intrusive access and multipoint installation. To suppress inverter switching noise as well as irradiance- and load-induced fluctuations, recent studies have adopted wavelet decomposition, resonant or band-pass filtering, differential features, and adaptive thresholding for real-time detection. In contrast, the radiated-measurement route enables non-contact deployment, which is naturally suitable for retrofit applications and large-area inspection, and can also be extended to localization and multi-sensor fusion.

Motivated by the above considerations, this paper focuses on the engineering objective of achieving non-contact, low-cost embedded, and cross-scenario robust arc-fault detection, and proposes a PV dc arc detection framework based on near-field magnetic coupling sensing and rule-lightweight AI fusion. Unlike most existing radiated- or conducted-feature extraction methods that rely on megahertz-level bandwidth, this work is based on experimental observations showing that arc events exhibit broadband energy elevation, and that a measurable and consistent energy increase remains observable within the 12–80 kHz sub-band. This finding makes it possible to constrain both the signal chain and the algorithm design to a low-frequency sub-band affordable for MCU-based implementation. To address background heterogeneity and narrowband interference, a self-learning robust normalization mechanism and single-frame shape constraints are introduced, enabling low-false-alarm real-time decision making on resource-constrained MCUs. The detailed definitions and parameter settings are presented in Section 2.

The main contributions of this paper are summarized as follows.

(1) Time-domain and time-frequency-domain evidence of broadband energy elevation for PV dc arc faults is provided, and the arc-related energy increase within the 12–80 kHz sub-band is quantitatively demonstrated.
(2) A robust normalized feature, denoted as Z(f), is proposed for heterogeneous field backgrounds, and its mechanism for distinguishing arc events from narrowband spikes is illustrated visually.
(3) A single-frame shape/coverage constraint is designed to reject narrowband interference, and a rule-AI cascaded decision strategy is further developed using a lightweight AI module, making the method suitable for implementation on resource-constrained MCUs.

## 2. Problem Formulation and Detection Pipeline

### 2.1. Signal Processing Paradigms

**Figure 1:**
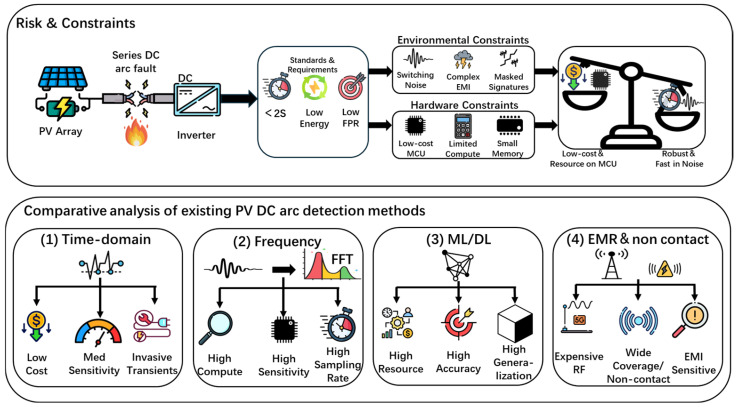
Comparative analysis of arc detection methodologies: schematic principles and qualitative trade-offs.

As illustrated in Figure 1, existing photovoltaic (PV) DC arc-fault detection techniques can be broadly categorized into four technical routes according to signal features and sensing modalities, each exhibiting a different trade-off among cost, computational burden, and robustness.

Time-domain methods are computationally lightweight and suitable for microcontroller implementation, but they usually rely on intrusive sensing and still face a fundamental trade-off between sensitivity and transient-induced false alarms [1,2,3]. Frequency-domain and time-frequency methods improve fault sensitivity by exploiting richer spectral information, yet their dependence on high sampling rates and computationally intensive transforms makes low-cost embedded deployment difficult [4,5,6]. Pure artificial-intelligence methods provide strong nonlinear discrimination capability, but their memory/computation demand, limited physical interpretability, and uncertain generalization under variable PV backgrounds remain important concerns for resource-constrained platforms [7,8,9,10]. Electromagnetic-radiation-based methods offer the key advantage of non-intrusive deployment, but many existing solutions still rely on expensive radio-frequency instrumentation or remain vulnerable to complex field interference [11,12,13,14].

Although existing studies have made progress at the theoretical level, PV dc arc-fault detection still faces three core challenges when practical engineering deployment is considered.

(1) Absence of engineering-grade non-contact sensing solutions

Mainstream arc-fault circuit interrupter (AFCI) products still rely heavily on intrusive current sensing, which implies wiring modification for existing PV systems, together with high retrofit cost and potential safety risks. Although EMR-based non-contact methods are theoretically attractive because of their plug-and-play nature, most reported solutions remain limited to expensive laboratory radio-frequency instruments [15,16,17], with little attention paid to embedded engineering design for compact spaces such as combiner boxes or the interior of microinverters.

(2) Mismatch between high-frequency sampling requirements and low-cost MCU constraints

Existing non-intrusive arc detection methods generally rely on megahertz-level sampling to capture transient radiation peaks. However, such high-speed data acquisition requirements are difficult to reconcile with the limited memory and processing power of low-cost microcontroller units (MCUs) [18,19].

To address these hardware constraints, the proposed framework focuses on the broadband power spectral density (PSD) uplift within a kHz-level sub-band (12–80 kHz). Instead of attempting to identify complex high-frequency patterns, our method detects arc anomalies by monitoring the overall spectral energy shift across this specific range. By leveraging this physical broadband characteristic, the system can achieve reliable arc discrimination with significantly lower sampling rates and a lightweight AI (MLP) model. This cascaded Rule–AI logic effectively minimizes the volume of data acquisition and computational overhead, enabling robust real-time protection on low-end edge devices without the need for expensive high-speed hardware.

(3) Cross-environment robustness and commissioning challenges in distributed PV scenarios

In real PV installations, wiring topology and electromagnetic background vary substantially across sites, and the noise amplitude may fluctuate by several tens of decibels. Conventional fixed-threshold methods often fail when transferred to new scenarios, resulting in frequent false alarms and repeated on-site tuning efforts. At present, an adaptive detection solution capable of achieving one-time configuration and multi-scenario applicability through a self-learning mechanism is still lacking.

**Figure 2:**
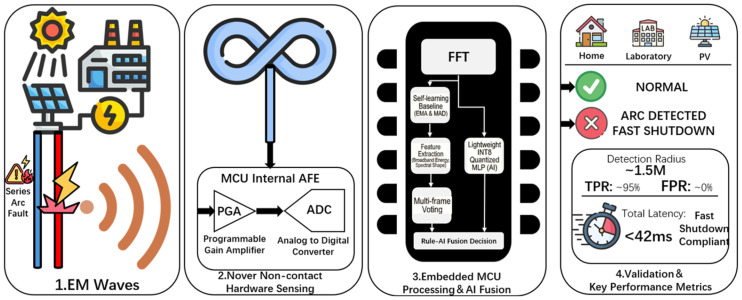
Graphical concept of the proposed method.

To address the above critical engineering gaps, this paper proposes an embedded nonintrusive detection system that integrates physical sensing, signal processing, and decision output into a unified framework. As shown in Figure 2, the core technical route of the proposed method forms a closed innovation loop consisting of four layers.

(1) Strategic sub-band selection

**Figure 3:**
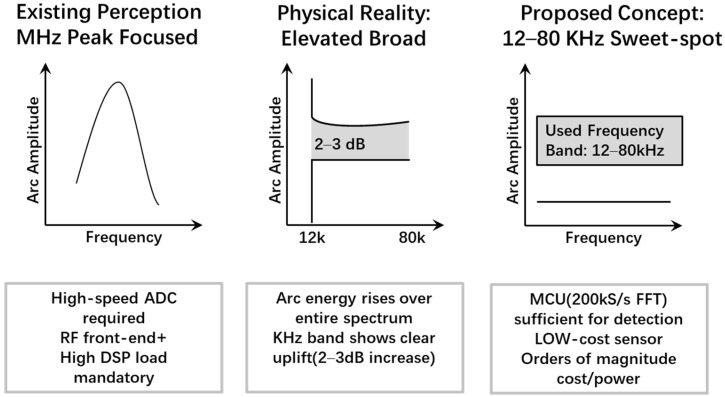
Graphical concept for the sub-band selection motivation.

Most existing studies focus on the analysis of the 42 MHz high-frequency band, where prominent arc spectral peaks are often observed. However, as illustrated in Figure 3, this work further reveals a key physical characteristic of arc events, namely broadband energy elevation over a wide frequency range. Both theoretical analysis and software-defined radio (SDR)-based experimental results indicate that even within the low-frequency sub-band of 12–80 kHz, arc faults still exhibit a significant signal gain. Based on this finding, an engineering sweet spot is established, in which accurate detection can be achieved using only the computational capability of a low-cost microcontroller unit (MCU), without relying on expensive high-performance digital signal processors (DSPs) or high-speed field-programmable gate arrays (FPGAs). This strategy fundamentally resolves the conflict between high-frequency sampling requirements and constrained embedded resources.

(2) High-sensitivity sensing construction

To match the above low-frequency strategy and enable efficient signal acquisition, the system introduces a coaxial shielded loop antenna based on Moebius topology. By directly coupling this sensor to the on-chip analog front end (AFE) of the MCU, the proposed design achieves high-signal-to-noise-ratio (SNR) acquisition in the 12–80 kHz band without requiring an external standalone amplifier, thereby substantially reducing hardware complexity and cost.

(3) Embedded processing and fused decision-making

On the resource-constrained MCU platform, an environment-adaptive baseline system based on the exponential moving average (EMA) is developed to dynamically decouple background noise. More importantly, to compensate for the limitations of conventional rule-based algorithms in ambiguous boundary cases, a lightweight INT8-quantized multilayer perceptron (MLP) is introduced. In this way, a robust hybrid decision architecture integrating rule-based logic and AI assistance is constructed, improving detection accuracy while preserving real-time performance.

(4) Cross-scenario end-to-end validation

Extensive experiments are conducted in multiple representative scenarios, including residential environments, laboratory setups, and real PV sites, as well as a particularly challenging weak-current condition of 2 A. The measured results strongly verify that the proposed system achieves high-sensitivity detection and an ultra-low false positive rate (FPR) close to 3% while maintaining very low hardware cost, thereby demonstrating its substantial practical value and reliability for real-world engineering applications.

### 2.2. Binary Decision Formulation and Evaluation Metrics

In this study, the arc discrimination problem is formulated as a binary hypothesis test with two classes: H0, corresponding to the absence of an arc (background or interference), and H1, corresponding to the presence of an arc (arc radiation). Under three representative environments, namely household, laboratory, and photovoltaic (PV) scenarios, the frequency-domain peak characteristics and temporal structure features under arc-free and arc-present conditions are compared to evaluate the stability of the proposed method across different backgrounds.

To assess detection performance, three metrics are adopted, namely the true positive rate (TPR), false positive rate (FPR), and decision latency. The TPR characterizes the probability of successfully detecting an arc event within a given sensing radius. The FPR represents the probability of false alarm occurrence per unit time under arc-free conditions. The decision latency measures the time elapsed from arc initiation to alarm generation by the system. Under the strict constraint of maintaining a low FPR, this work aims to maximize the achievable detection distance while controlling the decision latency, thereby satisfying the practical engineering requirements of low false-alarm rate, sufficient detection range, and rapid response.

### 2.3. Overview of the Detection Pipeline

**Figure 4:**
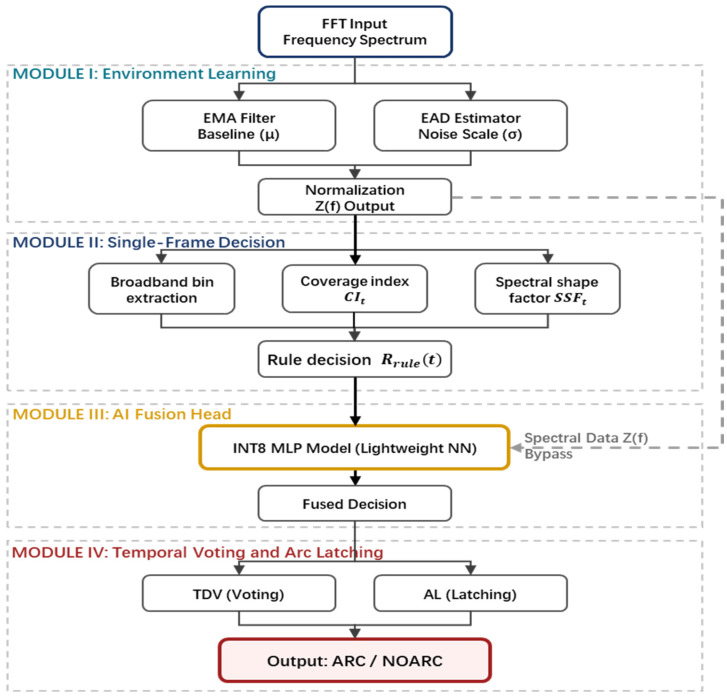
Overall signal-processing and decision pipeline of the proposed detector.

To achieve robust extraction of arc-related features under low-signal-to-noise-ratio (SNR) conditions, a hierarchical cascaded detection pipeline is constructed, as shown in Figure 4. This architecture consists of four core processing modules. Following the engineering sequence of baseline construction, preliminary feature screening, fused refined decision-making, and temporal stabilization, environmental interference is progressively suppressed and fault features are reliably identified.

Module I: Environment Learning

Because the background noise may drift significantly across different scenarios, it is difficult to directly determine whether a given signal corresponds to an arc event. To address this issue, this module performs online learning of the background mean and fluctuation scale at each frequency bin, and converts the spectrum of the current frame into a dimensionless anomaly measure, denoted by $Z_{t}(k)$. Both the subsequent rule-based and AI-based decision stages are built upon this module.

Let the input one-sided fast Fourier transform (FFT) spectrum of the t-th frame be $P_{t}(k)$ in dB, where the frequency-bin index k is restricted to the monitored sub-band from $k_{L}$ to $k_{H}$, as mapped from $f_{L}$ to $f_{H}$ (the corresponding parameters are listed in Table 1). For each frequency bin k, the background baseline $\mu_{t}\left(k\right)$ is recursively updated using the exponential moving average (EMA), as given in (1):

(1) $$\mu_{t}\left(k\right)=\left(1-\alpha \right)\mu_{t-1}\left(k\right)+\alpha P_{t}\left(k\right),$$

In (1), α denotes the EMA smoothing factor. The smoothing factor α controls the tracking speed of the background baseline. A smaller α leads to slower mean updating and lower sensitivity to transient arcing events or impulsive spikes, whereas a larger α allows faster adaptation to environmental drift but may also absorb abnormal components into the baseline. In this work, a fixed value of α is adopted (see Table 1) to balance slow-drift tracking against anomaly preservation.

To characterize the background fluctuation scale while improving robustness to outliers, a second-order variance is not used, since variance estimation is overly sensitive to environmental interference. Instead, an absolute-deviation estimator based on exponential sliding updates is introduced, referred to as the exponential absolute deviation (EAD) estimator. This estimator is used to describe the robust fluctuation scale of each frequency bin relative to the background mean, and its recursive form is given in (2):

(2) $$d_{t}\left(k\right)=\left(1-\alpha \right)d_{t-1}\left(k\right)+\alpha \left|P_{t}\left(k\right)-\mu_{t}\left(k\right)\right|,$$

In (2), the same α is used as the smoothing factor for the EAD update. Equation (2) replaces the squared error with the absolute deviation, thereby significantly reducing the influence of a single spike on the scale estimate. This makes the method more suitable for field environments with narrowband interference and transient disturbances. To avoid a zero denominator, a small constant ε is introduced (see Table 1).

(3) $$Z_{t}\left(k\right)=\frac{P_{t}\left(k\right)-\mu_{t}\left(k\right)}{d_{t}\left(k\right)+\varepsilon },$$

where $X_{t}(k)$ is the current spectral value, $\mu_{t}(k)$ is the updated background mean, $d_{t}(k)$ is the exponentially updated absolute deviation, α is the smoothing factor, and ε is a small constant introduced to avoid division by zero.

In this paper, the normalized frequency-domain anomaly statistic is generally denoted as Z(f). Considering that the practical system is implemented on the basis of discrete short-time spectra, it is expressed in engineering implementation as $Z_{t}(k)$, where t denotes the time-frame index and k denotes the discrete frequency-bin index within the monitored sub-band. Physically, $Z_{t}(k)$ represents the standardized elevation intensity of the current frame relative to the learned background at that frequency bin. Therefore, $Z_{t}(k)$ remains comparable under background shifts across different environments, such that subsequent modules only need to define unified thresholds and decision logic in the $Z_{t}(k)$ domain. The final normalized spectrum $Z_{t}(k)$ is then provided as the input to Module II (single-frame rule-based screening) and Module III (MLP-based decision).

Module II: Single-Frame Rule-Based Decision

Arc events typically exhibit broadband elevation over multiple frequency bins within the monitored sub-band, whereas environmental interference more commonly appears as narrowband carriers, in which only a few bins are significantly elevated, or as random spikes, in which isolated bins increase abruptly. Therefore, imposing broadbandness and continuity constraints on $Z_{t}(k)$ within a single frame can significantly reduce the false-alarm rate.

WB (wideband-elevation candidate set): As defined in (4), the set of elevated frequency bins is extracted using the per-bin anomaly threshold $\theta_{Z}$. This set transforms the single-frame spectrum from continuous amplitude values into a binary elevation representation, thereby facilitating subsequent broadbandness evaluation:

(4) $$B_{t}=\{k\in \left[k_{L},k_{H}\right]|Z_{t}\left(k\right)\geq \theta_{Z}\},$$

CI (coverage index): The CI is used to describe the proportion of frequency bins within the monitored band that are identified as elevated. Since narrowband interference usually affects only a small number of bins, the corresponding CI is typically low, whereas the broadband elevation induced by arc events leads to a larger CI.

(5) $$CI_{t}=\frac{\left|B_{t}\right|}{K},\;K=k_{H}-k_{L}+1,$$

SSF (spectral shape factor): To further suppress discrete pseudo-peaks, that is, occasional elevations occurring at multiple nonadjacent bins, the set $B_{t}$ is decomposed along the frequency axis into several contiguous runs. Let $L_{max}$ denote the number of bins in the longest contiguous run, as defined in (6). This quantity represents the maximum bandwidth of continuously elevated bins within a single frame. Narrowband carriers usually correspond to very narrow contiguous runs, while random spikes correspond to extremely short runs. In contrast, arc events tend to produce wider continuously elevated intervals.

(6) $$SSF_{t}=L_{max}\Delta f,\;\Delta f=F_{s}/N_{FFT},$$

(7) $$R_{rule}\left(t\right)=1\left(CI_{t}\geq \theta_{CI}\wedge SSF_{t}\geq \theta_{SSF}\right),$$

Module III: AI-Fusion Head

For boundary samples that are difficult to distinguish using rules alone, such as weak arcs or strong broadband interference, a lightweight INT8-quantized multilayer perceptron (MLP) is activated. This module takes the normalized single-frame spectrum $Z_{t}(k)$ as input and outputs the probability vector $P_{t}=[p_{0},p_{1}]$. To reduce computational overhead, the MLP is triggered only when the rule-based decision indicates non-arc while the sample remains in a gray zone, for example, when $R_{rule}(t)=0$ and either $CI_{t}$ or $SSF_{t}$ is close to its threshold. Otherwise, inference is skipped. Accordingly, samples that are directly classified as arc by the rule stack are accepted without invoking the MLP. The MLP is used only for rule-negative but boundary-adjacent samples, and can promote such samples to ARC when the predicted arc probability exceeds the decision threshold. The final fused decision is defined as:

(8) $$R_{fused}\left(t\right)=R_{rule}\left(t\right)\vee 1\left(p_{1}\geq \theta_{MLP}\right),$$

**Figure 5:**
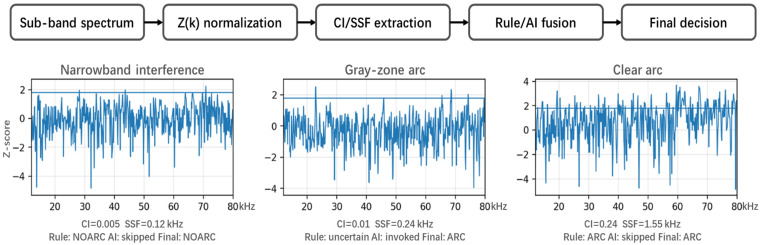
Representative Z-domain decision-path examples for narrowband interference, gray-zone arc, and clear arc cases in the proposed pipeline. The blue horizontal lines indicate the reference thresholds.

To make the decision path interpretable, Figure 5 presents representative Z-domain examples for narrowband interference, gray-zone arc, and clear arc cases, together with the extracted CI/SSF values and the corresponding Rule–AI decisions.

Module IV: Temporal Smoothing and State Latching

The single-frame fused decision $R_{fused}\left(n\right)$ may still produce false alarms due to narrowband interference. Therefore, this module takes $R_{fused}\left(n\right)$ in {0,1} as input, applies temporal decision voting over a sliding window of length L to perform majority-vote smoothing, and further introduces state latching with hysteresis and holding mechanisms to avoid frequent switching between the ARC and NOARC states. In this way, a stable system state is generated. The detailed strategy is presented in Section 5.

(9) $$V_{n}=\sum_{i=n-L+1}^{n}R_{fused}\left(i\right),$$

In (9), $V_{n}$ denotes the number of frames classified as arc faults within the current voting window, and L denotes the voting-window length. When $V_{n}$ satisfies the predefined consistency condition, the system enters or maintains the ARC state. Otherwise, once the exit condition is met, the system returns to the NOARC state.

## 3. Arc Physics and Engineering Frequency Band

### 3.1. Current Mutation and Radiation-Field Model

During arc ignition and sustained discharge, the current typically exhibits clustered pulsations and rapid transitions, leading to a significant increase in the time derivative $\frac{di_{arc}}{dt}$. In electromagnetic-radiation theory, the following expression is used to explain the mechanism by which current fluctuations give rise to broadband radiation:

(10) $$E_{arc}\left(t\right)=\frac{1}{4\pi \varepsilon_{00}c^{2}}\frac{di_{arc}}{dt},$$

Previous studies have reported spectral maxima in the tens-of-megahertz range (e.g., 40.6 MHz) under specific conditions [20,21]. However, as expressed in Equation (11), the peak frequency $f_{0}$ is highly sensitive to the equivalent inductance and dielectric environment of the discharge channel, and is therefore not universal:

(11) $$f_{0}\approx \frac{1}{2\pi \rho \varepsilon },$$

This peak drift supports the use of broadband in-band criteria (Z, CI, and SSF) rather than fixed-peak detection. Importantly, even when MCU hardware cannot directly acquire megahertz-level signals, arc-related low-frequency components remain observable due to the broadband nature of the plasma discharge. Combined with the frequency-weighting effect of the near-field magnetic loop, the 12–80 kHz sub-band becomes a viable “engineering sweet spot” for embedded detection.

**Figure 6:**
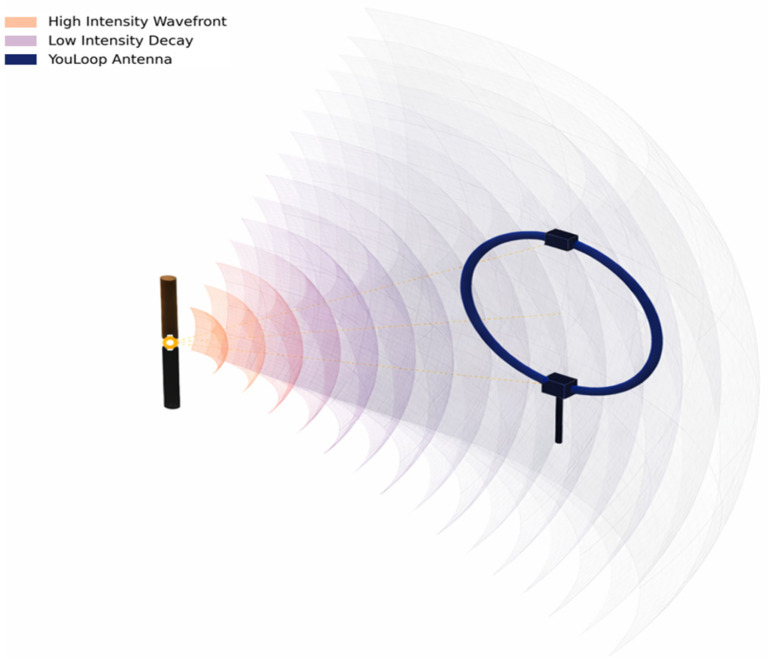
DC arc radiation propagation and near-field capture using a shielded loop antenna. The black bar denotes the arc source, and the blue loop denotes the antenna.

**Figure 7:**
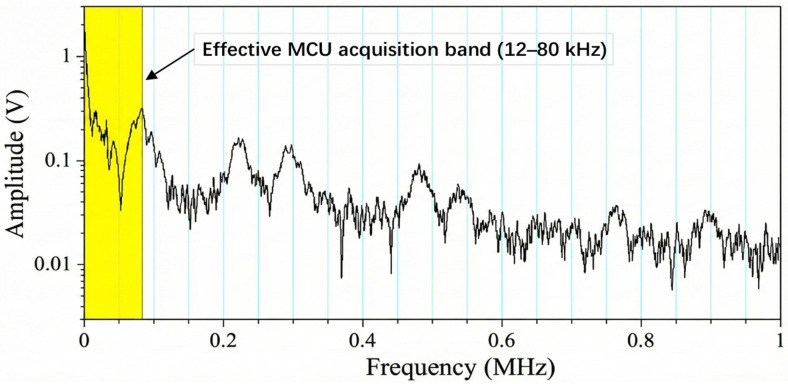
Amplitude spectrum of DC arc noise (0–1 MHz) with the selected 12–80 kHz engineering sub-band highlighted.

Figure 6 and Figure 7 illustrate the near-field capture concept and the selected engineering sub-band. In practical environments, stable narrowband interference—such as inverter switching harmonics and radio signals—often produces spectral spikes that can trigger false alarms in peak-based methods. By shifting the detection target from identifying a fixed peak to evaluating significant broadband anomalies within the monitored band, the proposed framework ensures robust interference immunity on resource-constrained platforms.

### 3.2. SDR Spectrum Analysis

Experiments on the arc generator were conducted under a 60 W load condition. The signals were acquired using a low-cost software-defined radio (RTL-SDR) receiver and visualized with spectrum-analysis software (SDR sharp v1770). The data shown in the figure correspond to a continuous 15 s recording. Owing to hardware limitations, the maximum observable bandwidth was 1.7 MHz. Within this range, typical arc characteristics can be observed. Compared with the background, arc events generally manifest themselves as energy elevation or enhanced statistical significance over a relatively wide frequency band, rather than as a peak increase at a single frequency point. Therefore, this paper does not use peak-frequency localization as the decision criterion, but instead evaluates whether the effective signal-to-noise ratio within the target operating band is sufficient for reliable detection.

**Figure 8:**
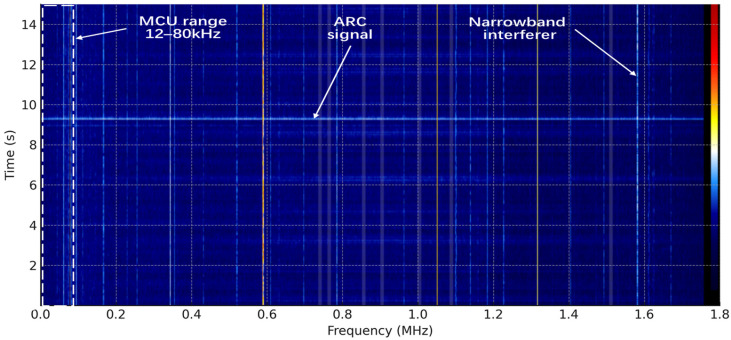
Household spectrogram (15 s; RTL-SDR Fs = 1.7 MHz; YL r = 1.0 m; NFFT 4096, Hann, hop 2048; dBFS). The right-side color bar indicates signal intensity, with blue representing lower intensity and yellow/red representing higher intensity.

As shown in Figure 8, the vertical lines correspond to steady narrowband interference continuously present throughout the 15 s measurement interval. Their different colors represent different signal strengths, as defined by the color scale on the right side of the figure. The horizontal features indicate moments at which energy rises across the full observed band. In contrast to narrowband interference, arc events usually appear as sustained energy enhancement spanning multiple frequency bins and multiple time frames. The box indicates the MCU detection range, from which it can be seen that the selected band covers the frequency region where arc activity is observed.

**Figure 9:**
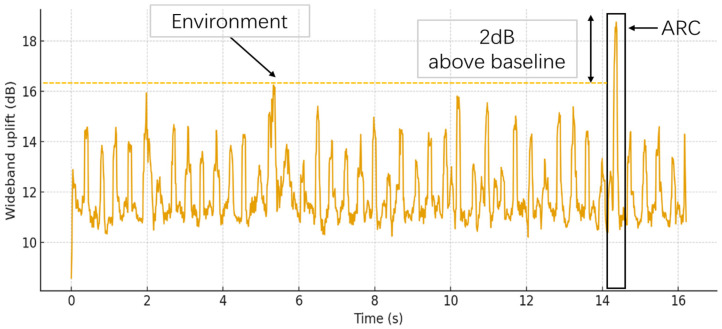
Time trajectory of the band-averaged spectral intensity in the selected 12–80 kHz engineering sub-band.

Figure 9 shows the time trajectory of the single-frame averaged spectral intensity within the 12–80 kHz engineering sub-band. This trajectory is obtained by compressing the spectral intensities of all frequency bins in the sub-band at each time instant into a single scalar, which is then stored as a single-index spectral-intensity curve. The results show that the environmental baseline, plotted in blue, remains stable within 0–16 dB, whereas the arc events, plotted in red, exhibit an elevation exceeding 18 dB. This finding demonstrates that even in the kilohertz band, arc signals can still be clearly distinguished from environmental noise.

**Figure 10:**
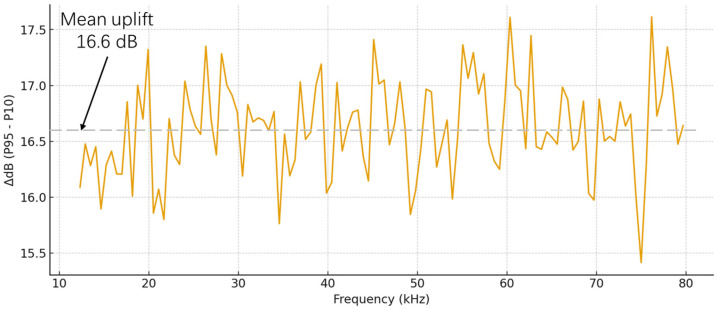
Statistical spectrum difference between arc and environment (household load, 12–80 kHz, N = 50, error bars: 95% CI).

To quantify the spectral difference between arc events and the background, 50 independent experiments were conducted. For each frequency bin, the 95th percentile and the 10th percentile of the energy distribution were calculated, and their difference was taken to obtain the ΔP curve, i.e., $P_{95}-P_{10}$. Specifically, for each frequency bin, the 95th percentile represents the arc-event level, whereas the 10th percentile represents the environmental baseline. Their difference therefore characterizes the uplift at that frequency bin. As shown in Figure 10, when an arc occurs, the average energy increase relative to the background reaches approximately 15–18 dB over the entire 12–80 kHz band. Here, the dB value refers to the inter-quantile difference at each frequency bin across multiple frames, based on the dB representation of the FFT amplitude spectrum. More specifically, the percentiles are computed separately for arc frames and background frames, and the uplift at each bin is defined using the 95th percentile of the arc frames and the 10th percentile of the background frames. This result visually confirms the broadband-elevation characteristic of arc events. Taken together with the theoretical analysis and the above figures, it can be concluded that the uplift within the 12–80 kHz range remains positive for most frequency bins. Although the maximum feature amplitude appears at the megahertz level, the kilohertz sub-band accessible on the MCU side still exhibits significant energy enhancement.

To more intuitively illustrate the time-domain difference between arc fault and the background, Figure 10 is provided. The upper panel, labeled Normal Operation (Background), shows a 20 ms time-domain waveform under background conditions. The thin blue trace represents the raw sampled fluctuation. Overall, the waveform mainly oscillates around zero. Although a small number of spikes are present, these spikes are sparse and nonconsecutive. The orange and green auxiliary lines represent the upper and lower amplitude bounds, respectively, providing a clear visualization of the signal excursion range and facilitating comparison with the arc condition. The root mean square reflects the overall fluctuation intensity, whereas the peak-to-peak value (p–p) reflects the maximum peak-to-valley excursion.

**Figure 11:**
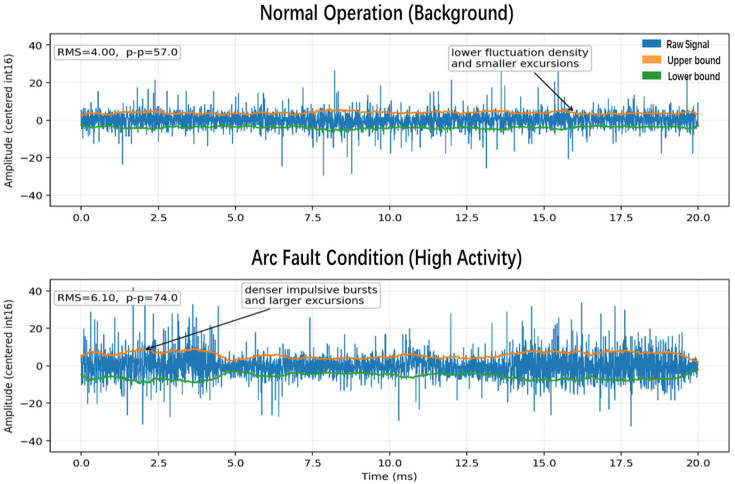
Representative time-domain waveforms extracted from a background window and an Arc Fault Condition window from raw.bin (20 ms, same vertical scale).

The lower panel in Figure 11, labeled Arc Fault Condition (High Activity), shows another 20 ms time-domain waveform exhibiting characteristics of an arc event. It can be observed that the blue waveform contains significantly more frequent and denser positive and negative spikes. Many of these spikes are clearly higher and deeper than those in the upper panel, indicating stronger instantaneous energy release during arc activity. In addition, both the root mean square and p–p values increase markedly. This demonstrates that, compared with the background segment, the arc segment exhibits denser impulsive fluctuations and larger instantaneous amplitude deviations, and therefore possesses stronger transient disturbance characteristics in the time domain.

### 3.3. Engineering Frequency Band

By jointly considering the on-chip analog-to-digital converter (ADC) sampling rate of the microcontroller unit (MCU), the available computational capability, and the bandwidth of the analog front-end amplifier, the monitored frequency band in this work is limited to 12–80 kHz. The lower bound of 12 kHz is mainly chosen to avoid the influence of power-frequency components and their low-order harmonics, low-frequency background fluctuations, and slowly varying disturbances. The upper bound of 80 kHz is selected to preserve a sufficient implementation margin under the current sampling rate, analog front-end bandwidth, and real-time spectral-processing capability, thereby avoiding an overly aggressive band choice close to the hardware limit. Under a sampling rate of approximately 200 kS/s and a 4096-point fast Fourier transform (FFT) configuration with a Hann window and a hop size of 2048, real-time operation can be achieved without overloading the MCU.

This choice does not contradict the conclusions in the literature regarding spectral peaks in the megahertz range. Through both theoretical analysis and SDR-based measurements, this paper shows that sufficient arc-related energy still exists within the 12–80 kHz sub-band. Combined with the approximately flat amplitude-frequency response of the front end in this band, as well as processing gains obtained from frame averaging and sub-band energy aggregation, stable arc detection can be achieved in this kilohertz-level sub-band, thereby striking a balance between feature informativeness and hardware complexity.

This paper does not claim that 12–80 kHz is the globally optimal frequency band for all scenarios. Rather, under the platform constraints considered in this work, it is adopted as an engineering compromise that balances immunity to low-frequency contamination, edge-side real-time capability, and implementation margin at the upper end of the spectrum.

## 4. MCU-Based Signal Processing and Decision Logic

### 4.1. Overall System Flow

**Figure 12:**
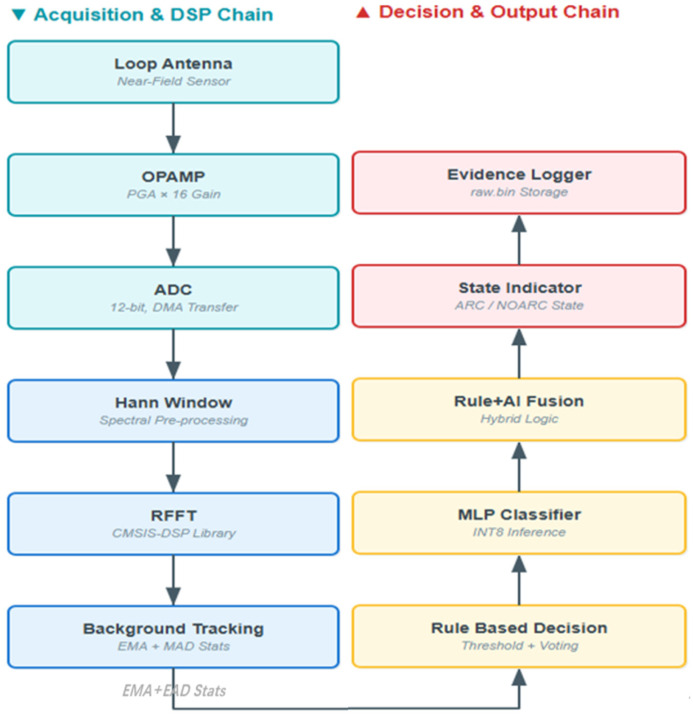
Implementation-oriented signal-processing and decision flow on the STM32H743 MCU.

Unlike architectures that rely on multistage sampling, communication, and cloud-based analysis, the complete detection chain in this work is consolidated onto a single microcontroller unit (MCU) platform [22]. As shown in Figure 12, the STM32H743-based system performs front-end signal acquisition, analog-to-digital converter (ADC) sampling, window preprocessing, real fast Fourier transform (RFFT)-based spectral transformation, online background-statistics updating, rule-based decision making, conditionally triggered multilayer perceptron (MLP)-assisted discrimination, rule–AI fusion, state indication, and evidence-log recording. This section focuses on the execution order and task allocation of the above modules along the MCU real-time path, without repeating the methodological definitions already presented in Section 2.

### 4.2. Sampling and Buffering

The front-end signal is acquired through the coaxial sensor and, after AC coupling and dc biasing, is fed into the on-chip operational amplifier OPAMP1 configured as a programmable gain amplifier (PGA) with a gain of 16, which directly drives the 12-bit ADC1. Additional details of the sensor and front-end design are provided in Appendix A. This gain setting provides the maximum achievable amplification within the available on-board range while ensuring that the ADC is not overloaded under the target arc-signal amplitude.

The ADC operates in continuous-conversion mode. Its sampling clock is derived from PLL2 division to approximately 6 MHz. With a configuration of 30 conversion clock cycles, the resulting effective sampling rate is approximately 200 kS/s. This sampling rate matches the subsequent spectral-analysis parameters, namely $N_{FFT}=4096$ and hop =2048, enabling full coverage of the monitored 12–80 kHz band while retaining sufficient anti-aliasing margin.

A circular direct memory access buffer is employed, with a total buffer length of 4096 samples (ADC_BUF_LEN=4096). Processing is performed in half-buffer units of 2048 samples: whenever one half of the buffer is filled, a spectral-analysis and decision procedure is triggered, while sampling continues on the other half. This double-buffer structure ensures parallel execution of acquisition and processing, thereby avoiding data loss and preventing operations such as SD-card writing from degrading real-time performance.

### 4.3. Spectral Computation

To effectively suppress spectral leakage while preserving frequency resolution within the monitored 12–80 kHz sub-band, the system performs a short-time Fourier transform based on a Hann window, with a window length of N=4096 and a hop size of 2048. A real fast Fourier transform (RFFT) and magnitude normalization are implemented using the ARM CMSIS-DSP library. After that, hard masking is applied to the dc and sub-low-frequency components, namely the first 10 frequency bins. This step eliminates the effects of baseline drift and sensor bias at the source, ensuring that the subsequent environment-learning and decision logic are driven only by valid ac arc-related features. Finally, the processed spectrum is converted to a logarithmic scale (dB) and passed to the background-modeling module.

### 4.4. Environment Learning and Background Modeling (EMA/EAD/Z)

To adapt to the long-term drift of background-noise amplitude in different photovoltaic (PV) scenarios, the system maintains a set of online-updated background statistics for each monitored frequency bin on the MCU side, and maps the current spectral value to a normalized anomaly quantity, denoted by Z. Considering the real-time and memory constraints of the STM32H743, an O(1)-complexity background tracker is constructed using the exponential moving average (EMA) and the exponential absolute deviation (EAD), so that stable online normalization can be achieved without introducing the storage overhead of a sliding window.

In practical deployment, the background level may differ by several tens of decibels across scenarios. If a fixed amplitude threshold is applied directly, the false-alarm rate can easily increase. Therefore, the system does not make decisions directly on the raw spectral amplitude. Instead, for each frequency bin, it separately maintains the background mean and fluctuation scale, and represents the current observation as a normalized deviation relative to the background. The background mean is updated recursively by the EMA, as given in (12):

(12) $$\mu \leftarrow \left(1-\alpha \right)\mu +\alpha P,$$

where P is the current spectral value at that frequency bin. The smoothing factor is set to α=0.05, corresponding to an effective time constant of approximately 200 frames, so as to preserve background-tracking capability under slow environmental drift while reducing the risk that strong transient events contaminate the background model.

To further improve statistical robustness, the system uses recursive EAD updating to maintain the absolute-deviation quantity for each frequency bin, thereby suppressing the influence of outliers on noise-floor estimation. The current spectral value is then mapped to the normalized anomaly quantity Z. By updating the background mean and fluctuation scale separately, the system can maintain consistent sensitivity to abnormal events under variable noise environments:

(13) $$Z=\frac{P-\mu }{EAD},$$

In addition, this modeling mechanism inherently provides a passive link self-diagnosis function. The algorithm continuously monitors the background energy floor within 12–80 kHz. If the updated baseline remains persistently far below the normal inactive-system range, indicating the loss of the expected coupled background, the system determines that sensor detachment or an open-circuit fault may have occurred.

Specifically, the detection logic focuses on the broadband energy uplift across the entire 12–80 kHz sub-band, rather than tracking specific narrowband peaks like inverter switching ripples. While environmental noises are typically periodic and narrowband, a DC arc manifests as a consistent rise in the overall power spectral density (PSD) floor. This approach ensures immunity against frequency-specific disturbances and allows the system to verify sensor integrity even during inactive periods (e.g., night) by monitoring the baseline thermal noise floor.

A significant advantage of this Z-domain normalization is its topology-agnostic robustness. Although arc events can typically be identified by their dominant broadband energy, the adaptive background modeling ensures consistent performance across various inverter architectures (e.g., string vs. micro-inverters) that exhibit different switching frequency profiles. By continuously tracking and normalizing frequency-specific stationary components, the system effectively suppresses narrowband harmonics regardless of the inverter type. This ensures that the decision logic remains focused on the stochastic broadband energy uplift characteristic of arc faults, providing a uniform detection sensitivity and high immunity against varying environmental noise levels.

### 4.5. Embedded Implementation of Spectral-Shape Criteria

After obtaining the normalized spectrum Z, spectral-shape constraints are further introduced in the frequency domain. Specifically, the spectral shape factor SSF (quality factor) and the coverage index CI are defined on the basis of above-threshold bins. The SSF jointly reflects the number of above-threshold bins within a frame, as well as their clustering and continuity over the monitored band, whereas the CI measures the effective continuous spectral coverage of these bins over the sub-band in terms of equivalent frequency width. When only one or two narrow peaks are present, both the SSF and the CI remain low. In contrast, when an arc event causes simultaneous energy elevation over the entire sub-band, both quantities increase significantly. Compared with methods relying only on a single-bin amplitude threshold, such shape-based criteria are less sensitive to absolute gain and calibration error, and are therefore more suitable for distributed deployment scenarios, although they require additional bin-level statistics on a frame-by-frame basis and proper bandwidth-threshold selection.

Empirical thresholds are defined on the normalized power spectrum: a triggering threshold of K=2.0 and a confirmation threshold of K=2.33, the latter approximately corresponding to the 98th percentile under arc-free conditions. As noted earlier, the dc and very-low-frequency bins are excluded from the decision process through front-end suppression and hard masking. The system then counts the number of above-threshold bins and computes the maximum contiguous bandwidth. A minimum bandwidth requirement of MIN_BIN_HZ=12 kHz is imposed, and the presence of a continuous elevated region satisfying BIG_BW_HZ≥48 kHz is used to distinguish arc-induced full-band elevation from narrowband interference.

### 4.6. Temporal Voting and Latching Mechanism

Single-frame spectral criteria alone remain susceptible to occasional spikes and narrowband interference. For example, in household experiments, a stable narrowband component generated by a Bluetooth speaker was observed to cause the entire test segment to be falsely classified as an arc event. To improve decision stability, the system introduces a temporal majority-voting and state-latching mechanism on top of the frequency-domain shape criteria. On the one hand, observations are accumulated and voted over multiple consecutive frames to suppress isolated false triggers. On the other hand, hysteresis and latching are used to maintain the alarm state, thereby avoiding frequent ARC/NOARC switching under marginal conditions.

In the normal mode, the system performs majority voting over a sliding window of N=4 frames. An arc alarm is issued only when at least K=2 frames simultaneously satisfy both the per-bin threshold and the broadband-consistency criteria. Release is controlled by an empirically determined hysteresis constant, Z_TAU_ = 2.7. This value was selected from multiple experimental trials to balance response speed against state oscillation, thereby providing a certain degree of stickiness when the decision variable is close to the threshold and preventing persistent state chattering.

Practical experiments show that stable narrowband interference is one of the major factors affecting decision stability. Therefore, when a frequency band is detected to contain a long-lasting stable narrowband component, characterized by a coefficient-of-variation threshold of CV_TH_GUARD = 0.15, the system enters an interference-guard mode. The entry and hold durations are set to 400 ms and 1500 ms, respectively. In this mode, stricter criteria are applied: the confirmation threshold is increased to K=2.58, and the temporal voting rule is changed to N=5,K=3. Once the guard mode is exited, the system returns to the normal threshold and window settings. In this way, false triggering caused by stable narrowband interference can be significantly reduced without changing the overall false-positive rate, at the cost of a moderate increase in detection latency.

### 4.7. AI-Assisted Decision and Fusion

**Figure 13:**
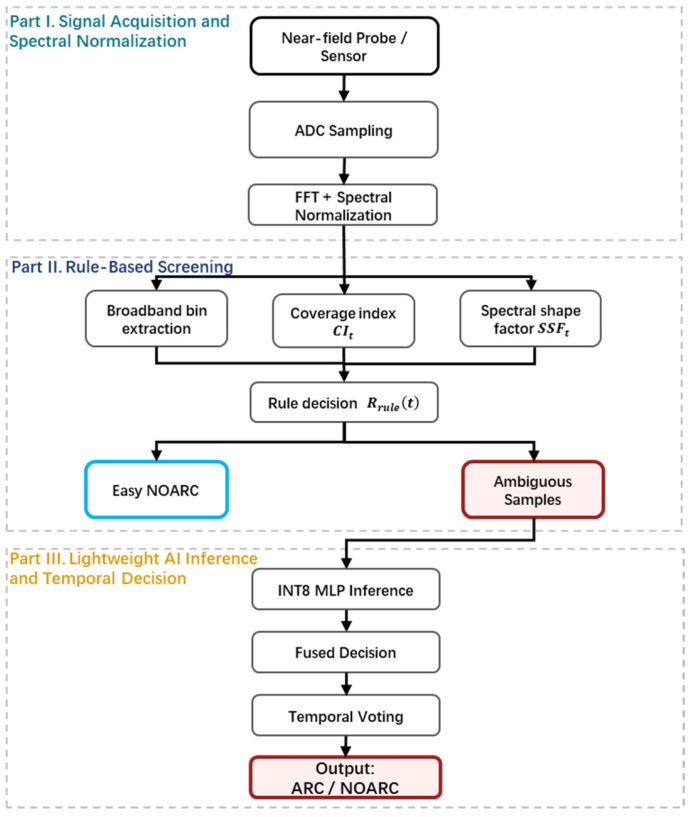
Conditional rule–AI refinement flow in the embedded detector.

To resolve the inherent trade-off between high sensitivity and an extremely low false positive rate (FPR), a hierarchical cascaded decision architecture is constructed, as shown in Figure 13. Through the conditional coupling of a two-stage decision logic, this architecture enables robust detection under complex boundary conditions while maintaining computational efficiency.

(1) Deterministic pre-screening

The first stage employs spectral-shape constraints as a primary filter to efficiently reject explicit interference such as inverter switching harmonics and background white noise. The logic at this stage is simple and highly responsive, and it is responsible for filtering out more than 90% of nonfault samples, thereby ensuring the fundamental stability of the system.

(2) Nonlinear auxiliary refinement

For ambiguous samples that cannot be reliably distinguished by linear rules alone, the system conditionally triggers a lightweight INT8-quantized multilayer perceptron (MLP). By exploiting the nonlinear fitting capability of the neural network, this module performs secondary confirmation near the decision boundary, thereby significantly improving the recall of weak arc features without sacrificing the overall false positive rate.

Under this architecture, the implementation and optimization of each functional module are described as follows.

(1) Dataset construction and block-wise splitting

To construct a high-fidelity feature space, the dataset integrates high-precision SDR recordings (approximately 1 GB) and embedded edge-side SD-card recordings (approximately 30 MB). After resampling and sliding-window segmentation, approximately 38,000 spectral-frame samples were generated.

To avoid data leakage caused by random splitting of time-series signals, a strict block-wise splitting strategy was adopted. Specifically, the first 80% of the temporal segments from each experimental recording were used as the training set, while the remaining 20% were used as a non-overlapping validation set. This strategy forces the model to learn the intrinsic spectral morphology of arc events rather than memorizing background-noise sequences, thereby ensuring that the evaluation results more faithfully reflect its generalization capability.

During preprocessing, the raw data were transformed into amplitude spectra through a 4096-point fast Fourier transform (FFT), followed by dB conversion and normalization. The final feature set $\left(X,y\right)$ was then constructed, where X denotes the normalized spectral frames within the 12–80 kHz sub-band.

(2) Lightweight model architecture and quantization

The AI module adopts a three-layer fully connected network, i.e., a multilayer perceptron (MLP). The input-layer dimension is matched to the number of effective FFT frequency bins, the hidden layer contains 128 rectified linear unit (ReLU) neurons, and the output layer performs binary classification between ARC and NOARC. After convergence under training in the Python environment using the Adam optimizer, the model is quantized to INT8 through the X-CUBE-AI toolchain by post-training quantization. This step compresses the model weights from FP32 to 8-bit integers, thereby significantly reducing storage and memory-access overhead and making the model compatible with the hardware-resource constraints of the STM32 platform.

(3) Embedded deployment and resource profiling

The quantized model contains approximately 262 k parameters. The Flash footprint is reduced from 1.0 MB in FP32 format to 256 kB, while the peak RAM usage during inference is controlled within 16–32 kB. Together with the preceding signal-processing chain, including the FFT and background statistics, the end-to-end latency of single-frame processing remains within 0.6–2.5 ms, and the average CPU utilization stays below 5%. Benefiting from the conditional triggering mechanism, the additional delay introduced by AI inference, approximately 2–10 ms, occurs only in a very small number of boundary cases. In this way, computational efficiency is maximized while embedded real-time requirements are still satisfied.

(4) Visualization of feature-space complementarity

**Figure 14:**
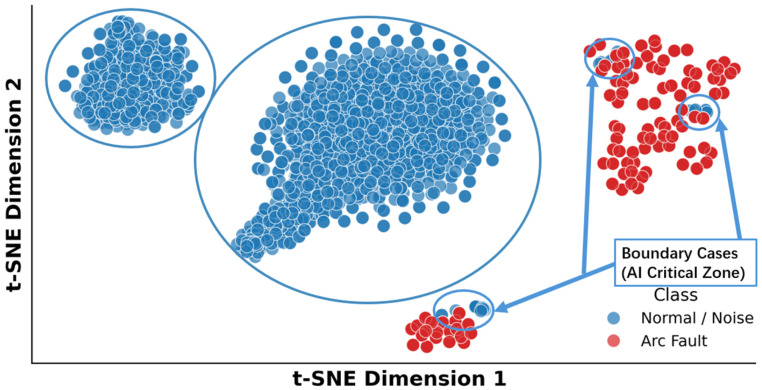
Visualization of feature distribution complementarity. The blue circles highlight representative feature clusters and boundary cases.

To visually verify the effectiveness of the fusion strategy, Figure 14 presents the t-distributed stochastic neighbor embedding (t-SNE) distribution of validation-set features after dimensionality reduction. The results show that background noise samples and steady arc samples exhibit significant separability in the feature space, forming two clearly distinguishable regions. By contrast, the samples marked as suspicious arcs by the rule stack are mainly distributed in the ambiguous transition zone between these two regions. Within this area, the AI module successfully constructs a nonlinear decision boundary and correctly classifies weak arc samples that would otherwise be missed by the rule-based detector, thereby demonstrating its critical auxiliary role in rule refinement under edge conditions.

### 4.8. Output and Evidence Chain

After the decision is completed, the system outputs the result through three channels: Liquid Crystal Display for immediate indication of the ARC/NOARC state, universal asynchronous receiver-transmitter logging, and SD-card storage in the form of a binary file, namely raw.bin. Among these, the SD-card output serves as the core evidence chain, recording spectral data and decision labels over a time interval before and after the occurrence of an arc event.

In terms of data structure, the storage format is unified as 16-bit signed integers (short int), strictly corresponding to the fast Fourier transform (FFT) magnitude values. To balance storage overhead against data integrity, the FFT results are stored after amplitude compression. Each frame contains 4096 sampled points, and 2049 int16 values are written per frame, corresponding to the one-sided real fast Fourier transform (RFFT) magnitude spectrum. The storage process is driven by direct memory access to ensure continuous and complete data acquisition.

**Figure 15:**
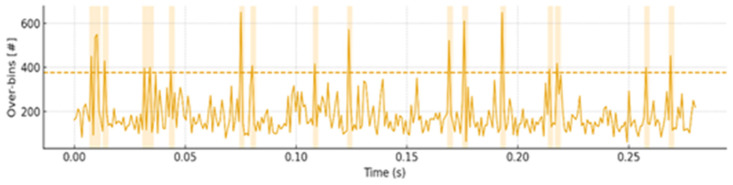
Over-threshold bin count versus time with adaptive threshold. The “#” symbol denotes the number of over-threshold bins, and the dashed line indicates the adaptive threshold.

Figure 15 shows the threshold-exceeding-bin-count versus time curve reconstructed from raw.bin, where the dashed line denotes the adaptive threshold. The quantization rule is given by int16 = round (amplitude × scale_k).

Within the system bandwidth of 12–80 kHz, the arc interval appears as a cluster of peaks, i.e., a clearly above-threshold feature. The arc segment is marked by the yellow shaded region, whereas the non-event region remains low and stable. These results indicate that the system can still reliably separate arc events from noise under complex background conditions, while also providing a numerically verifiable record for the evidence chain.

## 5. Experimental Setup and Data Acquisition

### 5.1. PV Field Scenario

**Figure 16:**
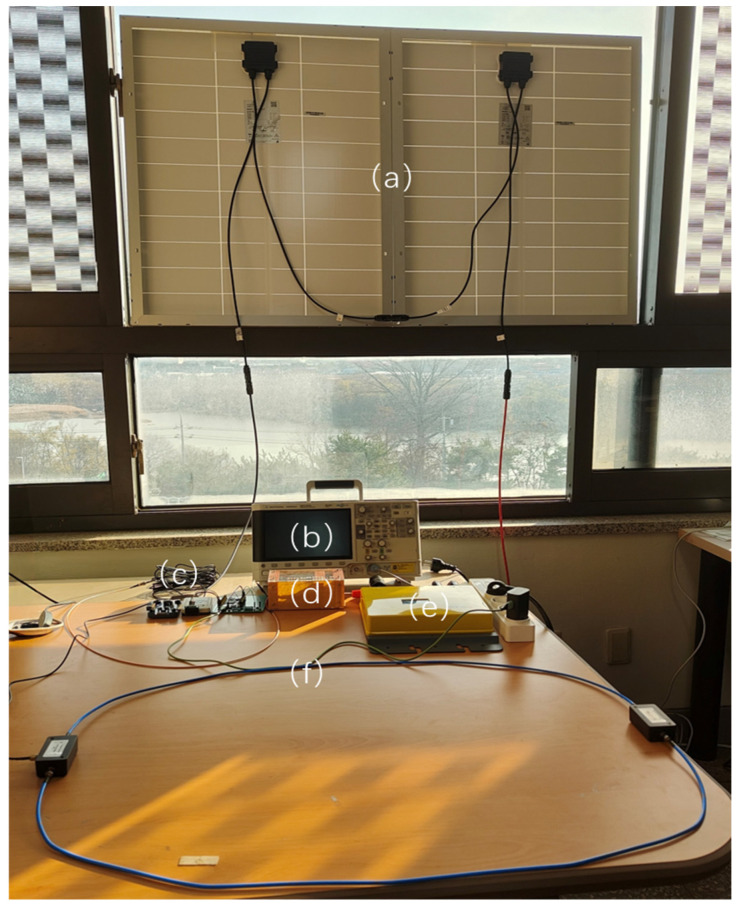
Experimental hardware setup for the PV field scenario: (**a**) two 100 W photovoltaic modules; (**b**) oscilloscope; (**c**) STM32H743 board and analog front-end; (**d**) manually operated arc generator; (**e**) 300 W grid-connected microinverter; (**f**) loop antenna placed on the table for near-field sensing.

A typical grid-connected microinverter-based photovoltaic (PV) system was constructed for the PV field experiment shown in Figure 16. In terms of hardware configuration, two 100 W PV modules (CSMS-100W-V2, Guangzhou Lvyuan Technology Co., Ltd., Guangzhou, China) were connected in series and interfaced to the power grid through a 300 W grid-connected microinverter (Powernet SOLAGE 300, model PIP102-300, Powernet, Seoul, Republic of Korea). In the figure, each module is highlighted and annotated using red boxes and text labels. The experiment was carried out by manually operating the arc generator, with an interval of approximately 1 s between successive operations.

To comprehensively evaluate the detection limit of the proposed system, the experiment was intentionally conducted under a weak-current boundary condition of 2 A (approximately 80 W). According to the electromagnetic-radiation model described in Section 3.1, this low-power condition represents a worst-case stress test, in which the arc radiation intensity is minimal and the detection difficulty is maximized. The experimental results show that even under such weak radiation conditions, the system can still accurately identify arc features, thereby fully demonstrating the high-SNR advantage of the near-field sensing chain.

In addition, for the typical high-current operating conditions of 10–20 A in large-scale PV plants, although the inverter switching noise is expected to increase significantly, the system retains sufficient dynamic-range margin to prevent ADC saturation by virtue of the reserved PGA gain rollback mechanism, which supports multilevel switching from 16× gain down to follower mode (i.e., unity gain). Combined with the previously described environment-learning mechanism and spectral-shape criteria, the system is capable of robust full-range detection from weak-current conditions to rated high-current operation.

### 5.2. Household Scenario

To introduce richer environmental factors and evaluate the cross-scenario adaptability of the proposed system, an ordinary household environment was selected as the second test scenario. In this setup, experiments were conducted separately using an adjustable dc power supply and a small PV module rated at 30 W, with the arc generator connected in series. The household environment contains various switching power supplies and domestic appliances, which produce continuous electromagnetic-interference noise. Experimental observations show that, without the environment-learning function, the false-alarm rate in this scenario increases significantly. This scenario therefore provides a comparative benchmark for evaluating the false-alarm suppression effect of the proposed environment-learning mechanism and baseline dB-elevation criterion.

### 5.3. Laboratory Scenario

During the multi-scenario false-alarm analysis, it was found that, in addition to household appliances, the school laboratory environment also exhibited a relatively high false-alarm rate. Multiple instruments operate continuously in the laboratory, and the superposition of their switching power supplies and communication modules forms a more complex electromagnetic-noise background. These interferences usually appear as narrowband spectral lines persisting at several fixed frequencies, and occasionally multiple frequency bins rise simultaneously within a short time interval. In this scenario, an adjustable dc power supply and a series-connected arc generator were again used to perform series-arc experiments. By using the environment-learning mechanism and baseline dB-elevation criterion proposed earlier, the system responds only to sudden broadband elevation relative to the environmental baseline, thereby effectively suppressing false alarms caused by the above persistent narrowband noise.

### 5.4. Spectrogram Recordings and SD-Based Dataset

While performing real-time arc decision-making on the MCU, the system simultaneously records sampled data near the arc event onto the SD card for offline spectrogram visualization and observation. During offline analysis, the same fast Fourier transform (FFT) and decision pipeline as that used in the embedded implementation is reused on the PC side in Python (3.12). In the plotted figures, only the arc intervals are highlighted in yellow to facilitate comparison with the background noise.

**Figure 17:**
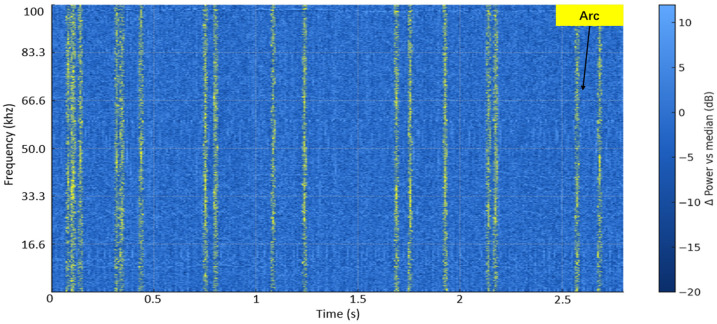
Household environment spectrum with arcs highlighted in yellow.

Figure 17 shows a representative SD-recorded spectrogram from the household scenario. In this spectrogram, the blue region corresponds to the background, while the yellow-highlighted interval marks the arc event. The experiments were conducted using an adjustable dc power supply and a 30 W PV panel as the power sources, while household devices such as a Wi-Fi router, a Bluetooth speaker, a microwave oven, and a laptop computer were operating simultaneously. It can be seen that the background remains relatively clean, whereas clear broadband-elevation stripes still appear in the 0–1.2 MHz range when the arc is triggered. The yellow highlighted region corresponds to a single arc event, thereby providing representative samples for subsequent evaluation of environment learning and false-alarm suppression.

### 5.5. Response Latency and Timing Analysis

In this work, the detection latency is defined as the time interval from the onset of the arc event, i.e., the instant at which the electromagnetic (EM) indicator exhibits a significant jump, to the output of the arc decision by the device. The decision strategy follows the majority-voting scheme described in Table 2 indicates that the dominant latency contribution comes from the hop-based temporal voting logic (~37 ms), whereas the computation term (~6 ms) already includes FFT-based processing, rule evaluation, and conditional AI inference.

IEC 63027 specifies explicit response-time verification requirements for dc series arc faults in photovoltaic (PV) systems. In addition, industry materials related to UL 1699B commonly cite an interruption limit of less than 2 s, under the constraint that the arc energy should not exceed 750 J. Under the voting-based decision logic adopted in this work, the maximum algorithm-side detection latency is bounded within 37 ms.

At the system-integration level, a high-speed control interface is reserved to drive commercial PV dc solid-state relays or rapid shutdown device. The typical physical response time of such devices is approximately 5 ms. Therefore, the theoretical total clearing time of the proposed system is about 42 ms. This value corresponds to only 2.1% of the 2000 ms upper limit commonly referenced in practice, indicating that, after integration with standard interruption hardware, the proposed system provides sufficient timing margin under the tested assumptions for PV rapid-shutdown applications.

## 6. Experimental Results and Discussion

Under the same dataset and threshold settings, the four modules in the detection chain were selectively enabled and disabled to quantify the contributions of different combinations to the true positive rate (TPR) and false positive rate (FPR), namely wideband elevation (WB), environment-learning-based baseline criterion (EL), exponential moving average smoothing (EMA), and lightweight artificial intelligence assistance (AI). Across the three scenarios (Home/Lab/PV), the cumulative monitoring time was approximately 50 h, while the arc-active intervals accounted for only about 1–2 min in total. Under this evaluation, the overall detection rate was approximately 97%, whereas the remaining 3% mainly arose from failed ignition or non-sustained arc attempts. The corresponding TPR/FPR results are summarized in Table 3.

Rapid environmental transients, such as short-duration irradiance fluctuations or temporary background spikes, are mitigated by the combined action of environment-adaptive baseline learning, EMA smoothing, temporal voting, latching, and the interference-guard logic.

When only the wideband-elevation (WB) rule is used, narrowband interference such as dc power-supply ripple, switching-power-supply noise, and Wi-Fi/Bluetooth emissions in the household scenario, as well as inverter switching ripple in the PV scenario, can all lead to false triggering, causing the system to remain in a persistently high false-alarm state.

After the environment-learning (EL) baseline rule is introduced, the system successfully detects all 30 standard arc events in the three scenarios. Here, “standard arc events” refer to valid arc events that develop into clearly visible and sustained combustion. As a result, a high detection rate for standard arc events is achieved. It should be noted that if arc attempts that fail to develop into clearly visible and sustained combustion after contact separation of the arc generator are also included in the statistics, the overall detection rate is approximately 97%, and the FPR is significantly reduced from nearly 100% to approximately 2–5%. This result indicates that the EL mechanism restricts the decision basis to broadband energy elevation relative to a dynamic baseline, thereby effectively suppressing irrelevant environmental noise. On this basis, the introduction of EMA smoothing further reduces threshold jitter and improves the temporal consistency of the decision. During the PV test window, the final configuration achieved an overall detection rate of 97%, with the remaining 3% mainly associated with failed ignition or non-sustained arc attempts under the tested conditions.

After lightweight AI is introduced, the conservative rule stack, which is designed to guarantee an extremely low false positive rate, still exhibits an inherent detection blind zone under sub-threshold conditions. In this context, the AI module provides nonlinear compensation and improves decision stability for boundary samples without increasing the observed false alarms. In this work, the denominator of the TPR is the number of standard arc events, excluding micro-arcs and failed ignition attempts, whereas the FPR is defined as the time ratio, i.e., false-alarm duration divided by the total monitoring time.

**Figure 18:**
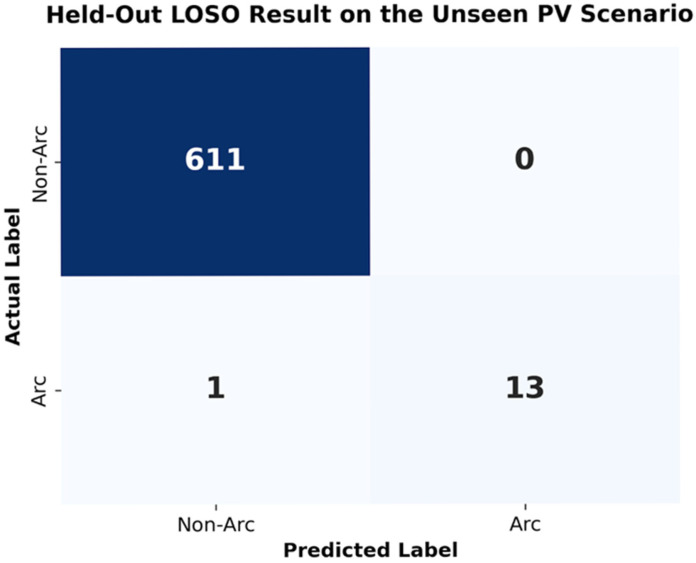
Confusion matrix of the held-out LOSO test on the unseen PV scenario, where the lightweight AI model was trained on the Home/Lab scenarios and directly tested on the previously unseen PV dataset without any scene-specific retraining.

To further evaluate the system’s adaptability to unseen environments, a Leave-One-Scenario-Out (LOSO) test was conducted. The lightweight AI model was trained exclusively using data from the Home/Lab scenarios and then directly tested on the noisy PV field environment without any scene-specific retraining. As shown in Figure 18, the fixed AI model achieved a TPR of 92.86% on the previously unseen PV dataset, while the residual error level remained at approximately 3% under the tested conditions.

The present validation is mainly based on the tested microinverter configuration. Since the proposed method relies on environment-adaptive Z-domain normalization rather than a fixed inverter-specific spectral peak, broader validation on other inverter topologies is left for future work.

## 7. Conclusions and Future Work

This paper presented a fully embedded, non-contact arc detection system based on near-field magnetic coupling and rule-AI fusion, specifically designed for early warning in photovoltaic (PV) dc systems. By employing a shielded small loop as a non-intrusive sensor and leveraging the MCU on-chip OPAMP-ADC-FFT signal chain, the proposed system enables efficient signal acquisition and spectral analysis within the 12–80 kHz sub-band. On this basis, a hierarchical decision framework composed of wideband elevation (WB), environment learning (EL), EMA smoothing, and a lightweight INT8-quantized multilayer perceptron (MLP) was constructed, achieving unified detection capability across household, laboratory, and PV scenarios.

Experimental results demonstrated that the final configuration (WB + EL + EMA + AI) achieved a 100% true positive rate for standard visible sustained arc events in the tested scenarios, while the overall detection rate was approximately 97% when failed ignition and non-sustained arc attempts were included. The remaining 3% was mainly associated with unsuccessful arc establishment or short-lived arc events under the tested conditions, and the total system response latency remained below 42 ms.

Compared with conventional approaches that rely on intrusive series-current sensing or megahertz-level high-sampling-rate analysis, the proposed method offers three main engineering advantages.

(1) Non-intrusive deployability: The sensing terminal operates in a fully non-contact manner, enabling deployment inside combiner boxes or microinverters without modification of the main dc circuit.
(2) Resource-efficient real-time processing: By shifting the detection band to a kilohertz-level sub-band and exploiting the processing gains provided by near-field magnetic coupling and frame averaging, real-time detection can be achieved on low-power, low-cost MCUs with limited sampling capability.
(3) Low-commissioning potential: Through EMA/EAD-based background modeling and spectral-shape constraints, the thresholds and model parameters showed stable behavior across the tested scenarios, suggesting reduced recalibration effort under similar deployment conditions.

It should be noted that the present work focuses on non-intrusive fault detection and embedded deployability rather than spatial fault localization. Compared with localization-oriented non-intrusive methods, the proposed framework prioritizes low-cost real-time warning under strict embedded hardware constraints. Accordingly, future work will focus on field validation under higher power levels and a wider variety of PV inverter topologies, as well as the extension of the proposed framework toward multi-sensor fault localization and comparison with localization-oriented approaches. In addition, the proposed detection module will be integrated with dc circuit breakers or electronic switches to form a complete arc-fault detection and interruption link compatible with IEC/UL requirements. Its extension to emerging dc applications, such as energy-storage dc buses and dc fast-charging systems, will also be explored.

## Figures and Tables

**Table 1:** Key Parameters of the Detection Pipeline.

Parameter	Description	Value
*F_s_*	Sampling frequency (Hz)	200,000 (200 kS/s)
*N_FFT_*	Number of FFT points	4096
*N_hop_*	Frame hop size (samples)	2048
*Window*	Window function and frame length	Hann, length 4096
*[f_L_, f_H_]*	Monitored sub-band (Hz)	[12,000, 80,000]
Δ*f*	Frequency resolution (Hz)	F_s_/N_FFT_ ≈ 48.8 Hz
*α*	EMA smoothing factor	0.05
*ε*	Division-protection constant in (3)	1 × 10^−9^
*θ_Z_*	Per-bin exceedance threshold in (4)	2.70
*θ_CI_*	Coverage threshold	0.02 (k)
*θ_SSF_*	Shape/continuity threshold	30,000 Hz
*L*	Voting-window length (frames)	5 (3-out-of-5 voting)

$R_{rule}(t)$, together with the interpretable intermediate variables $CI_{t}$ and $SSF_{t}$, which are used for Module III fusion and Module IV temporal smoothing.

**Table 2:** Experimental platform and sampling parameters.

Item	Time (ms)	Description
Single-frame duration	20.5	4096/200 kS/s
Hop interval	10.3	2048/200 kS/s
Computation time	6	FFT + band-energy extraction + rule-based decision + MLP
FFT chain	3	included in the computation time
3/5 voting decision	37	hop interval × 3 + computation time
Actuation response	5	relay switching time
Total time	42	theoretical interruption time

The above table shows the system-level timing from fault occurrence to circuit interruption. The detection time corresponds to the maximum measured value. The actuation response is referenced from the datasheet parameters of a typical industrial dc solid-state relay.

**Table 3:** TPR/FPR of different module combinations in three scenarios (detection side only). TPR is reported for standard visible sustained arc events.

Scene	Method	TPR	FPR
Home/PV/Lab	WB	–	100%
Home	WB + EL	93%	4%
Lab	WB + EL	97%	2%
PV	WB + EL	97%	5%
PV	WB + EL + EMA	93%	0%
Home	WB + EL + EMA + AI	97%	0%
PV	WB + EL + EMA + AI	97%	3%
Lab	WB + EL + EMA + AI	97%	0%

Under the WB-only scheme, almost all frames are classified as arc, and therefore the TPR is not meaningful for comparison and is denoted by “–”.

**Figure A1:**
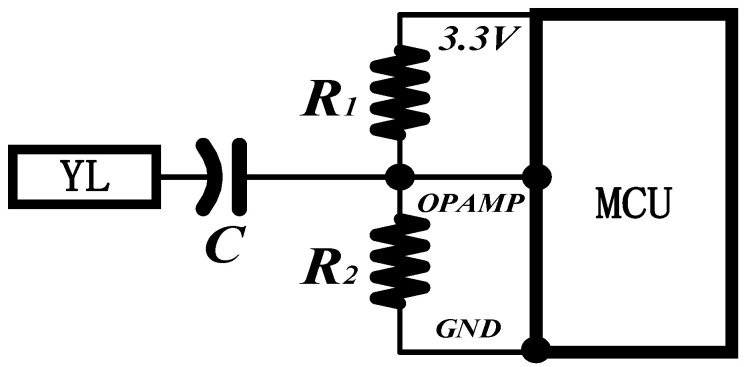
Front-end AC coupling and DC bias interface circuit for the MCU OPAMP.

**Figure A2:**
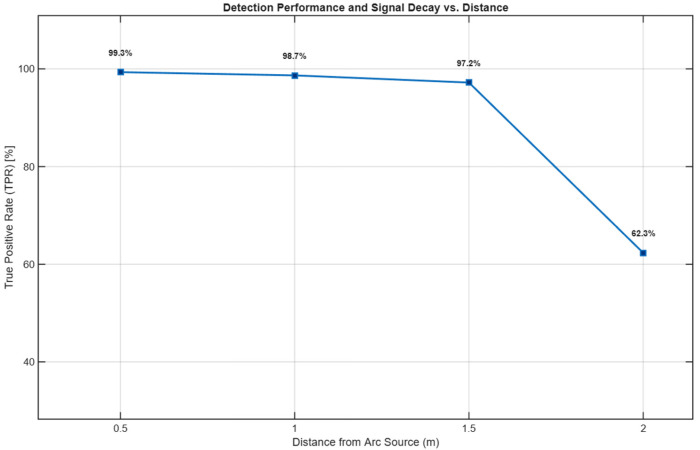
Empirical TPR versus sensing distance, together with the expected near-field magnetic decay trend.

## Data Availability

The raw data supporting the conclusions of this article will be made available by the authors upon request.

## Appendix A. Sensor and Front-End Design

This section establishes and analyzes two types of near-field sensors, namely the coaxial Moebius loop (YL) and the telescopic monopole. The objective is to provide a repeatable and stable coupling solution for arc-related near-field signals within the monitored 12–80 kHz band, and to explain the differences between the two sensors in terms of amplitude stability, signal-to-noise ratio (SNR), and environmental sensitivity.

### Appendix A.1. Antenna Modeling and Comparison

To overcome the limitations of conventional loop antennas in complex near-field electromagnetic environments, this work adopts a passive magnetic loop based on Moebius topology, namely the YouLoop (YL). The physical number of turns is N=1 (with an equivalent induction capability of 2), and the loop radius is a (30 cm). The effective area can be approximated as $A\approx N\pi a^{2}$. By constructing an electrically balanced structure through crossed interconnection of the coaxial-cable shield, effective suppression of electric-field interference is achieved within the monitored 12–80 kHz band under near-field quasi-static conditions (ka<1, r<λ), thereby establishing a magnetic-dominant coupling mode. Figure A1 shows the geometric structure of the coaxial shielded loop antenna used in this study, where the red arrows indicate representative field directions for near-field magnetic coupling.

Since the input impedance of the front end is much higher than the loop impedance, the sensor can be regarded as an ideal voltage source. The induced voltage $V_{YL}$ and the external magnetic field satisfy Faraday’s law of electromagnetic induction:

(A1) $$V_{\mathrm{YL}}\left(j\omega \right)=-j\omega \mu_{0}NAH_{⊥}\left(j\omega \right),$$

where $\mu_{0}$ is the permeability of free space. As can be seen from (A1), the YL exhibits a strictly linear response to both the angular frequency ω and the magnetic-field strength, and this broadband linear characteristic provides a predictable physical basis for subsequent signal-feature extraction.

In the near-field region, the arc source can be approximated as a dipole, whose magnetic-field magnitude decays with distance according to an inverse-cubic law:

(A2) $$H\left(r,\omega \right)\propto \frac{1}{r^{3}},$$

This inverse cubic decay implies an approximate 18 dB amplitude reduction for each doubling of distance, providing an engineering basis for the SNR boundary and gain-compensation requirement of the back-end analog front end.

### Appendix A.2. Front-End AC Coupling and DC Biasing

To satisfy the signal-acquisition constraints of an MCU-based single-supply system, a passive analog front-end interface based on an RC network is designed in this work.

As shown in Figure A1, the circuit employs a 100 nF coupling capacitor together with a 20 k\Omega/1 k\Omega resistor divider to form a signal path that provides both level shifting and high-pass filtering. The coupling capacitor and the equivalent parallel resistance of the divider network form a first-order high-pass response, whose cutoff frequency is located near the power frequency and its low-order harmonics. As a result, the circuit can effectively block the dc component and suppress interference below 12 kHz.

Together with the on-chip programmable gain amplifier (PGA) configured with a gain of 16, the static output operating point of the signal chain is shifted to 2.4 V. Under a 3.3 V full-scale range, this bias level reserves approximately 0.9 V of upper headroom, thereby providing the necessary linear swing margin for large dynamic burst signals generated during arc events. In this way, complete acquisition of weak bipolar signals can be achieved without requiring a negative supply rail. In addition, a PGA gain fallback mechanism, reducing the gain to 8 when necessary, is reserved to mitigate the potential risk of saturation under strong-coupling conditions.

### Appendix A.3. Engineering Applicability and Interference Immunity

In the experimental configuration of this study, the YL was primarily tested in a horizontally placed orientation, under which stable detection results were obtained. Experimental results indicate that this structure is not highly sensitive to installation orientation. Therefore, effective sensing can be achieved without strict angular alignment.

Applicable range: The proposed sensor is intended for operation in the near-field region, with a recommended deployment radius of 2.0 m. Repeated tests show that, under the current experimental conditions, stable detection performance can be maintained within approximately 1.5 m. An additional TPR-versus-distance analysis showed that the empirical TPR remained at 97% within 1.5 m, but decreased to 62% at 2.0 m, supporting 1.5 m as the maximum reliable detection radius under the tested conditions. Beyond this distance, the detection capability gradually degrades under the combined influence of multiple factors.

Installation clearance: Large metallic objects, strongly coupled conductors, and similar interference sources should be avoided within 20 cm of the sensor, since they may alter the near-field distribution and consequently degrade the decision signal-to-noise ratio (SNR) of the system. Small-amplitude motion disturbances in field deployment can be absorbed by the downstream noise-floor tracking, voting, and latching mechanisms.

Orientation and polarization: The most stable spectral pattern is obtained when the detector and the target arc are approximately aligned in the same direction within the horizontal plane. Under this condition, the spectral features become more distinct. If the sensor deviates from the optimal orientation, the detection performance may decrease and the stability of the decision criteria may be affected. In practical deployment, installation tolerances and slight oscillations may introduce coupling fluctuations; these slow-varying and short-duration disturbances are handled by the downstream noise-floor tracking, voting, and latching mechanisms.

Edge-side system bandwidth: The terminal-side system bandwidth is set to 12–80 kHz. This setting is derived from the computational constraints of the MCU and the environmental survey results. If environmental changes make the 12 kHz lower bound no longer stable, the main monitored band can be reselected according to the optimization equations provided later in this paper.

Main interference sources: Extensive experiments were carried out in three types of environments. The main interference sources include inverter switching harmonics, household appliance EMI, broadcast/shortwave leakage, occasional discharges, and local motor sparking. Feature analysis shows that such interference sources usually exhibit narrowband or intermittent distributions in the vicinity of 12 kHz, which remain distinguishable from arc events in terms of sub-band energy coverage and time-frequency morphology.

On-chip suppression capability: The on-chip processing chain can suppress most short-duration narrowband interference. The embedded decision logic includes mechanisms for excluding steady-state interference sources and learning the environmental background during system startup. When the false-alarm rate is tightly constrained but the recall becomes insufficient, the MLP-based rule-fusion mechanism can be enabled to improve detectability under boundary conditions.
